# 2,2-Bis(4-but­oxy­phen­yl)-1,1,1-trichloro­ethane

**DOI:** 10.1107/S1600536812032680

**Published:** 2012-07-25

**Authors:** Graham Smith

**Affiliations:** a Science and Engineering Faculty, Queensland University of Technology, GPO Box 2434, Brisbane, Queensland 4001, Australia

## Abstract

In the structure of the title compound, C_22_H_27_Cl_3_O_2_, which is the 4-but­oxy­phenyl analogue of the insecticidally active 4-meth­oxy­phenyl compound meth­oxy­chlor, the dihedral angle between the two benzene rings is 79.61 (11)°. Present also in the structure is an intra­molecular aromatic C—H⋯Cl inter­action.

## Related literature

For background to the mode of action of DDT analogues, see: Läuger *et al.* (1944 ▶); Kennard & Smith (1980 ▶). For the structures of the insecticides DDT and meth­oxy­chlor, see: DeLacy & Kennard (1972 ▶); Smith *et al.* (1976 ▶).
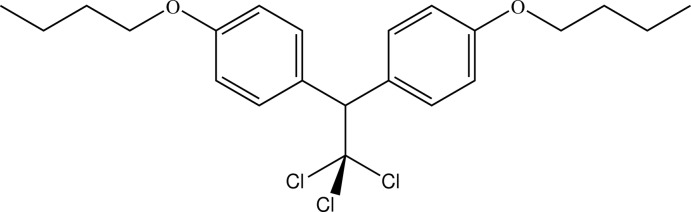


## Experimental

### 

#### Crystal data

C_22_H_27_Cl_3_O_2_
*M* *_r_* = 429.79
Monoclinic, 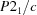

*a* = 5.7871 (1) Å
*b* = 18.3112 (5) Å
*c* = 20.3988 (4) Å
β = 91.160 (2)°
*V* = 2161.19 (8) Å^3^
*Z* = 4
Mo *K*α radiation
μ = 0.44 mm^−1^
*T* = 200 K
0.15 × 0.15 × 0.10 mm

#### Data collection

Oxford Gemini-S CCD area-detector diffractometer
Absorption correction: multi-scan (*CrysAlis PRO*; Agilent, 2012 ▶) *T*_min_ = 0.951, *T*_max_ = 0.981
14149 measured reflections
4245 independent reflections
3486 reflections with *I* > 2σ(*I*)
*R*_int_ = 0.035

#### Refinement

*R*[*F* ^2^ > 2σ(*F* ^2^)] = 0.047
*wR*(*F* ^2^) = 0.110
*S* = 1.07
4245 reflections
244 parameters
H-atom parameters constrained
Δρ_max_ = 0.35 e Å^−3^
Δρ_min_ = −0.18 e Å^−3^

### 

Data collection: *CrysAlis PRO* (Agilent, 2012 ▶); cell refinement: *CrysAlis PRO*; data reduction: *CrysAlis PRO*; program(s) used to solve structure: *SIR92* (Altomare *et al.*, 1993 ▶); program(s) used to refine structure: *SHELXL97* (Sheldrick, 2008 ▶) within *WinGX* (Farrugia, 1999 ▶); molecular graphics: *PLATON* (Spek, 2009 ▶); software used to prepare material for publication: *PLATON*.

## Supplementary Material

Crystal structure: contains datablock(s) global, I. DOI: 10.1107/S1600536812032680/bt5980sup1.cif

Structure factors: contains datablock(s) I. DOI: 10.1107/S1600536812032680/bt5980Isup2.hkl

Supplementary material file. DOI: 10.1107/S1600536812032680/bt5980Isup3.cml

Additional supplementary materials:  crystallographic information; 3D view; checkCIF report

## Figures and Tables

**Table 1:** Hydrogen-bond geometry (Å, °)

*D*—H⋯*A*	*D*—H	H⋯*A*	*D*⋯*A*	*D*—H⋯*A*
C2*B*—H2*B*⋯Cl3	0.93	2.69	3.361 (2)	130

**Fig. 1.**
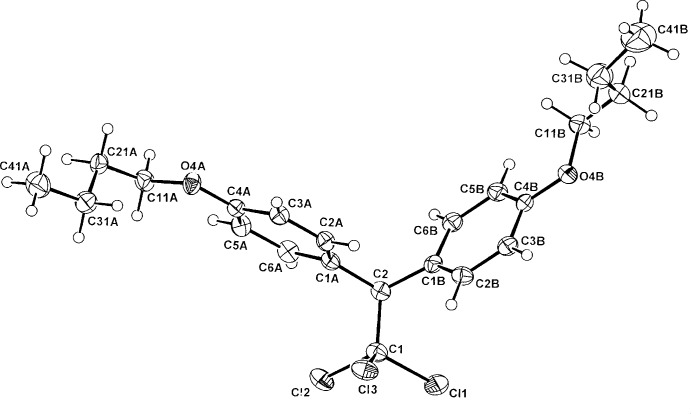
Molecular conformation and atom numbering scheme for the title compound, with displacement ellipsoids drawn at the 40% probability level.

**Fig. 2.**
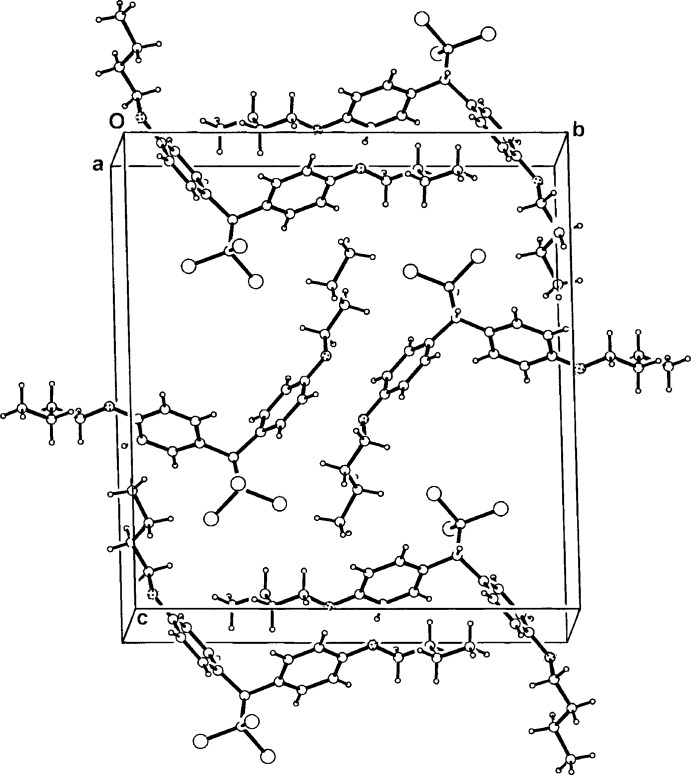
A perspective view of the crystal packing in the unit cell viewed down a.

## supplementary crystallographic information

### Comment

The title compound, C_22_H_27_Cl_3_0_2_, is the 4-butoxyphenyl analogue of the insecticide DDT [1,1,1-trichloro-2,2-bis(4-chlorophenyl)ethane] as well as the 4-methoxyphenyl analogue, also an insecticide (methoxychlor). Criteria for insecticidal activity of the DDT analogues have been described (Läuger *et al.*, 1944; Kennard & Smith, 1980). The crystal structures of both DDT (DeLacy & Kennard, 1972) and methoxychlor (Smith *et al.*, 1976) have been reported but there are no other structures of 4-alkoxyphenyl DDT derivatives in the crystallographic literature.

In the structure of the title compound (Fig. 1), the dihedral angle between the two phenyl planes is 79.61 (11)° which compares with 77.7° in the structure of methoxychlor (Smith *et al.*, 1976) and 64.7° in DDT (DeLacy & Kennard, 1972). The conformations of the two butoxy side chains relative to their phenyl rings (*A* and *B*) are essentially identical [comparative torsion angles C3—C4—O4—C11, C4—O4—C11–C21, O4—C11—C21—C31 and C11—C21—C31—C41 are -173.0 (2), 167.44 (19), -62.3 (3), -177.8 (2)° (*A*) and -170.0 (2), 168.5 (2), -63.9 (3), -171.1 (3)° (*B*), respectively]. The *B* ring conformation is stabilized by an intramolecular aromatic C2*B*—H···Cl3 interaction (Table 1). Present in the crystal packing is a relatively short intermolecular Cl···Cl contact [3.4302 (9) Å] but no other significant interactions are found (Fig. 2).

### Experimental

The title compound was obtained as an analytical reference standard from the US Public Health Service. Colourless crystal blocks suitable for X-ray analysis were obtained by room temperature evaporation of a solution in ethanol.

### Refinement

Hydrogen atoms were included in the refinement at calculated positions [C—H = 0.93–0.98 Å, with *U*_iso_(H) = 1.2*U*_eq_(C)(aromatic, methylene and methine) or 1.5*U*_eq_(C)(methyl), using a riding-model approximation.

### Crystal data

**Table:**

C_22_H_27_Cl_3_O_2_	*F*(000) = 904
*M**_r_* = 429.79	*D*_x_ = 1.321 Mg m^−^^3^
Monoclinic, *P*2_1_/*c*	Melting point = 321–323 K
Hall symbol: -P 2ybc	Mo *K*α radiation, λ = 0.71073 Å
*a* = 5.7871 (1) Å	Cell parameters from 4362 reflections
*b* = 18.3112 (5) Å	θ = 3.2–28.8°
*c* = 20.3988 (4) Å	µ = 0.44 mm^−^^1^
β = 91.160 (2)°	*T* = 200 K
*V* = 2161.19 (8) Å^3^	Block, colourless
*Z* = 4	0.15 × 0.15 × 0.10 mm

### Data collection

**Table:**

Oxford Gemini-S CCD area-detector diffractometer	4245 independent reflections
Radiation source: Enhance (Mo) X-ray source	3486 reflections with *I* > 2σ(*I*)
Graphite monochromator	*R*_int_ = 0.035
Detector resolution: 16.077 pixels mm^-1^	θ_max_ = 26.0°, θ_min_ = 3.2°
ω scans	*h* = −7→7
Absorption correction: multi-scan (*CrysAlis PRO*; Agilent, 2012)	*k* = −22→22
*T*_min_ = 0.951, *T*_max_ = 0.981	*l* = −23→25
14149 measured reflections	

### Refinement

**Table:**

Refinement on *F*^2^	Primary atom site location: structure-invariant direct methods
Least-squares matrix: full	Secondary atom site location: difference Fourier map
*R*[*F*^2^ > 2σ(*F*^2^)] = 0.047	Hydrogen site location: inferred from neighbouring sites
*wR*(*F*^2^) = 0.110	H-atom parameters constrained
*S* = 1.07	*w* = 1/[σ^2^(*F*_o_^2^) + (0.0482*P*)^2^ + 0.8549*P*] where *P* = (*F*_o_^2^ + 2*F*_c_^2^)/3
4245 reflections	(Δ/σ)_max_ < 0.001
244 parameters	Δρ_max_ = 0.35 e Å^−^^3^
0 restraints	Δρ_min_ = −0.18 e Å^−^^3^

### Special details

**Table:**

Geometry. Bond distances, angles *etc*. have been calculated using the rounded fractional coordinates. All su's are estimated from the variances of the (full) variance-covariance matrix. The cell e.s.d.'s are taken into account in the estimation of distances, angles and torsion angles
Refinement. Refinement of *F*^2^ against ALL reflections. The weighted *R*-factor *wR* and goodness of fit *S* are based on *F*^2^, conventional *R*-factors *R* are based on *F*, with *F* set to zero for negative *F*^2^. The threshold expression of *F*^2^ > σ(*F*^2^) is used only for calculating *R*-factors(gt) *etc*. and is not relevant to the choice of reflections for refinement. *R*-factors based on *F*^2^ are statistically about twice as large as those based on *F*, and *R*-factors based on ALL data will be even larger.

### Fractional atomic coordinates and isotropic or equivalent isotropic displacement parameters (Å^2^)

**Table:**

	*x*	*y*	*z*	*U*_iso_*/*U*_eq_	
Cl1	0.48915 (12)	0.34506 (4)	0.74543 (3)	0.0482 (2)	
Cl2	0.56526 (11)	0.19339 (4)	0.77193 (3)	0.0427 (2)	
Cl3	0.11570 (10)	0.24145 (4)	0.72992 (3)	0.0414 (2)	
O4A	0.2523 (3)	−0.03999 (8)	0.55790 (8)	0.0337 (5)	
O4B	0.2658 (3)	0.45228 (9)	0.44987 (8)	0.0366 (5)	
C1	0.4168 (4)	0.25582 (13)	0.71850 (11)	0.0314 (7)	
C1A	0.4329 (4)	0.16819 (12)	0.62234 (11)	0.0270 (6)	
C1B	0.4276 (4)	0.30454 (11)	0.59910 (11)	0.0269 (6)	
C2	0.4927 (4)	0.24406 (12)	0.64762 (11)	0.0284 (7)	
C2A	0.2207 (4)	0.15300 (12)	0.59082 (11)	0.0288 (7)	
C2B	0.2214 (4)	0.34358 (12)	0.59692 (11)	0.0320 (7)	
C3A	0.1674 (4)	0.08342 (12)	0.56947 (11)	0.0285 (7)	
C3B	0.1743 (4)	0.39339 (12)	0.54757 (11)	0.0311 (7)	
C4A	0.3241 (4)	0.02695 (12)	0.57889 (11)	0.0279 (7)	
C4B	0.3318 (4)	0.40494 (11)	0.49815 (11)	0.0286 (7)	
C5A	0.5381 (4)	0.04132 (13)	0.60786 (12)	0.0327 (7)	
C5B	0.5411 (4)	0.36850 (12)	0.50109 (11)	0.0332 (7)	
C6A	0.5886 (4)	0.11156 (12)	0.62884 (12)	0.0325 (7)	
C6B	0.5860 (4)	0.31939 (12)	0.55096 (11)	0.0310 (7)	
C11A	0.3962 (4)	−0.10180 (12)	0.57314 (12)	0.0345 (7)	
C11B	0.4096 (5)	0.45527 (14)	0.39336 (12)	0.0404 (8)	
C21A	0.2577 (4)	−0.16968 (12)	0.55871 (12)	0.0353 (8)	
C21B	0.2872 (5)	0.49745 (15)	0.34070 (13)	0.0474 (9)	
C31A	0.0447 (4)	−0.17793 (14)	0.59953 (13)	0.0411 (8)	
C31B	0.0676 (5)	0.46320 (17)	0.31572 (15)	0.0584 (11)	
C41A	−0.0850 (5)	−0.24830 (15)	0.58550 (15)	0.0555 (10)	
C41B	−0.0314 (7)	0.5020 (2)	0.25494 (19)	0.0901 (16)	
H2	0.66190	0.24500	0.65010	0.0340*	
H2A	0.11420	0.19040	0.58420	0.0340*	
H2B	0.11320	0.33600	0.62930	0.0380*	
H3A	0.02580	0.07440	0.54870	0.0340*	
H3B	0.03620	0.41940	0.54740	0.0370*	
H5A	0.64650	0.00420	0.61310	0.0390*	
H5B	0.65140	0.37710	0.46950	0.0400*	
H6A	0.73280	0.12090	0.64800	0.0390*	
H6B	0.72770	0.29540	0.55230	0.0370*	
H11A	0.44370	−0.10070	0.61900	0.0410*	
H11B	0.44190	0.40620	0.37810	0.0480*	
H12A	0.53360	−0.10090	0.54670	0.0410*	
H12B	0.55530	0.47870	0.40470	0.0480*	
H21A	0.35610	−0.21190	0.56590	0.0420*	
H21B	0.39060	0.50350	0.30430	0.0570*	
H22A	0.21120	−0.16930	0.51280	0.0420*	
H22B	0.25210	0.54570	0.35740	0.0570*	
H31A	−0.05760	−0.13690	0.59100	0.0490*	
H31B	−0.04590	0.46440	0.35010	0.0700*	
H32A	0.08970	−0.17660	0.64560	0.0490*	
H32B	0.09680	0.41240	0.30510	0.0700*	
H41A	−0.21810	−0.25090	0.61270	0.0830*	
H41B	−0.17280	0.47860	0.24120	0.1350*	
H42A	0.01440	−0.28920	0.59470	0.0830*	
H42B	0.07790	0.49940	0.22020	0.1350*	
H43A	−0.13350	−0.24940	0.54020	0.0830*	
H43B	−0.06130	0.55220	0.26520	0.1350*	

### Atomic displacement parameters (Å^2^)

**Table:**

	*U*^11^	*U*^22^	*U*^33^	*U*^12^	*U*^13^	*U*^23^
Cl1	0.0668 (4)	0.0431 (4)	0.0344 (3)	−0.0102 (3)	−0.0077 (3)	−0.0077 (3)
Cl2	0.0421 (3)	0.0535 (4)	0.0324 (3)	−0.0020 (3)	−0.0047 (3)	0.0116 (3)
Cl3	0.0317 (3)	0.0603 (4)	0.0324 (3)	−0.0021 (3)	0.0060 (2)	0.0022 (3)
O4A	0.0360 (9)	0.0260 (8)	0.0385 (9)	0.0029 (7)	−0.0114 (7)	0.0006 (7)
O4B	0.0472 (10)	0.0334 (9)	0.0294 (9)	0.0045 (7)	0.0049 (8)	0.0043 (7)
C1	0.0288 (11)	0.0377 (13)	0.0276 (12)	−0.0036 (10)	−0.0033 (10)	0.0003 (10)
C1A	0.0239 (11)	0.0311 (11)	0.0262 (11)	−0.0030 (9)	0.0027 (9)	0.0033 (9)
C1B	0.0284 (11)	0.0252 (11)	0.0270 (11)	−0.0071 (9)	−0.0008 (9)	−0.0051 (9)
C2	0.0239 (11)	0.0325 (12)	0.0287 (11)	−0.0032 (9)	0.0003 (9)	0.0013 (10)
C2A	0.0265 (11)	0.0299 (12)	0.0298 (12)	0.0034 (9)	−0.0025 (9)	0.0040 (10)
C2B	0.0288 (12)	0.0381 (13)	0.0292 (12)	−0.0021 (10)	0.0063 (10)	0.0004 (10)
C3A	0.0242 (11)	0.0327 (12)	0.0284 (12)	−0.0009 (9)	−0.0049 (9)	0.0017 (10)
C3B	0.0286 (11)	0.0322 (12)	0.0327 (12)	0.0016 (9)	0.0030 (10)	−0.0035 (10)
C4A	0.0306 (12)	0.0280 (11)	0.0252 (11)	−0.0004 (9)	0.0002 (9)	0.0020 (9)
C4B	0.0362 (12)	0.0229 (11)	0.0267 (11)	−0.0048 (9)	−0.0011 (10)	−0.0033 (9)
C5A	0.0258 (11)	0.0342 (13)	0.0378 (13)	0.0036 (10)	−0.0035 (10)	0.0041 (10)
C5B	0.0355 (12)	0.0340 (12)	0.0305 (12)	−0.0028 (10)	0.0089 (10)	−0.0012 (10)
C6A	0.0223 (11)	0.0383 (13)	0.0369 (13)	−0.0027 (9)	−0.0026 (10)	0.0031 (11)
C6B	0.0292 (12)	0.0312 (12)	0.0326 (12)	0.0003 (9)	0.0022 (10)	−0.0030 (10)
C11A	0.0340 (12)	0.0343 (13)	0.0352 (13)	0.0057 (10)	−0.0023 (10)	0.0053 (10)
C11B	0.0507 (15)	0.0379 (13)	0.0329 (13)	−0.0036 (12)	0.0093 (12)	−0.0008 (11)
C21A	0.0439 (14)	0.0260 (12)	0.0359 (13)	0.0054 (10)	−0.0034 (11)	0.0011 (10)
C21B	0.0691 (19)	0.0395 (14)	0.0340 (14)	0.0006 (13)	0.0078 (13)	0.0041 (12)
C31A	0.0454 (15)	0.0387 (14)	0.0389 (14)	−0.0053 (11)	−0.0031 (12)	−0.0008 (11)
C31B	0.064 (2)	0.0591 (19)	0.0518 (18)	0.0070 (15)	−0.0039 (15)	−0.0013 (15)
C41A	0.0625 (18)	0.0533 (17)	0.0504 (17)	−0.0198 (14)	−0.0050 (15)	0.0034 (14)
C41B	0.105 (3)	0.098 (3)	0.066 (2)	0.027 (2)	−0.031 (2)	−0.012 (2)

### Geometric parameters (Å, º)

**Table:**

Cl1—C1	1.771 (2)	C2—H2	0.9800
Cl2—C1	1.787 (2)	C2A—H2A	0.9300
Cl3—C1	1.782 (2)	C2B—H2B	0.9300
O4A—C4A	1.361 (3)	C3A—H3A	0.9300
O4A—C11A	1.436 (3)	C3B—H3B	0.9300
O4B—C4B	1.361 (3)	C5A—H5A	0.9300
O4B—C11B	1.436 (3)	C5B—H5B	0.9300
C1—C2	1.535 (3)	C6A—H6A	0.9300
C1A—C2	1.519 (3)	C6B—H6B	0.9300
C1A—C2A	1.402 (3)	C11A—H11A	0.9700
C1A—C6A	1.378 (3)	C11A—H12A	0.9700
C1B—C2	1.527 (3)	C11B—H11B	0.9700
C1B—C2B	1.391 (3)	C11B—H12B	0.9700
C1B—C6B	1.384 (3)	C21A—H21A	0.9700
C2A—C3A	1.379 (3)	C21A—H22A	0.9700
C2B—C3B	1.382 (3)	C21B—H21B	0.9700
C3A—C4A	1.386 (3)	C21B—H22B	0.9700
C3B—C4B	1.389 (3)	C31A—H31A	0.9700
C4A—C5A	1.387 (3)	C31A—H32A	0.9700
C4B—C5B	1.383 (3)	C31B—H31B	0.9700
C5A—C6A	1.385 (3)	C31B—H32B	0.9700
C5B—C6B	1.379 (3)	C41A—H41A	0.9600
C11A—C21A	1.505 (3)	C41A—H42A	0.9600
C11B—C21B	1.490 (4)	C41A—H43A	0.9600
C21A—C31A	1.509 (3)	C41B—H41B	0.9600
C21B—C31B	1.497 (4)	C41B—H42B	0.9600
C31A—C41A	1.516 (4)	C41B—H43B	0.9600
C31B—C41B	1.530 (5)		
			
C4A—O4A—C11A	118.04 (18)	C6A—C5A—H5A	120.00
C4B—O4B—C11B	116.51 (19)	C4B—C5B—H5B	120.00
Cl1—C1—Cl2	107.05 (12)	C6B—C5B—H5B	120.00
Cl1—C1—Cl3	108.74 (13)	C1A—C6A—H6A	119.00
Cl1—C1—C2	110.56 (16)	C5A—C6A—H6A	119.00
Cl2—C1—Cl3	106.56 (12)	C1B—C6B—H6B	119.00
Cl2—C1—C2	109.97 (16)	C5B—C6B—H6B	119.00
Cl3—C1—C2	113.68 (16)	O4A—C11A—H11A	110.00
C2—C1A—C2A	121.9 (2)	O4A—C11A—H12A	110.00
C2—C1A—C6A	120.8 (2)	C21A—C11A—H11A	110.00
C2A—C1A—C6A	117.4 (2)	C21A—C11A—H12A	110.00
C2—C1B—C2B	126.4 (2)	H11A—C11A—H12A	108.00
C2—C1B—C6B	116.4 (2)	O4B—C11B—H11B	110.00
C2B—C1B—C6B	117.2 (2)	O4B—C11B—H12B	110.00
C1—C2—C1A	112.36 (19)	C21B—C11B—H11B	110.00
C1—C2—C1B	115.93 (19)	C21B—C11B—H12B	110.00
C1A—C2—C1B	113.05 (18)	H11B—C11B—H12B	108.00
C1A—C2A—C3A	121.1 (2)	C11A—C21A—H21A	109.00
C1B—C2B—C3B	121.3 (2)	C11A—C21A—H22A	109.00
C2A—C3A—C4A	120.3 (2)	C31A—C21A—H21A	109.00
C2B—C3B—C4B	120.5 (2)	C31A—C21A—H22A	109.00
O4A—C4A—C3A	115.7 (2)	H21A—C21A—H22A	108.00
O4A—C4A—C5A	124.7 (2)	C11B—C21B—H21B	109.00
C3A—C4A—C5A	119.6 (2)	C11B—C21B—H22B	109.00
O4B—C4B—C3B	116.3 (2)	C31B—C21B—H21B	109.00
O4B—C4B—C5B	124.9 (2)	C31B—C21B—H22B	109.00
C3B—C4B—C5B	118.8 (2)	H21B—C21B—H22B	108.00
C4A—C5A—C6A	119.3 (2)	C21A—C31A—H31A	109.00
C4B—C5B—C6B	119.9 (2)	C21A—C31A—H32A	109.00
C1A—C6A—C5A	122.4 (2)	C41A—C31A—H31A	109.00
C1B—C6B—C5B	122.3 (2)	C41A—C31A—H32A	109.00
O4A—C11A—C21A	107.73 (18)	H31A—C31A—H32A	108.00
O4B—C11B—C21B	108.9 (2)	C21B—C31B—H31B	109.00
C11A—C21A—C31A	114.4 (2)	C21B—C31B—H32B	109.00
C11B—C21B—C31B	114.6 (2)	C41B—C31B—H31B	109.00
C21A—C31A—C41A	112.8 (2)	C41B—C31B—H32B	109.00
C21B—C31B—C41B	112.5 (3)	H31B—C31B—H32B	108.00
C1—C2—H2	105.00	C31A—C41A—H41A	109.00
C1A—C2—H2	105.00	C31A—C41A—H42A	109.00
C1B—C2—H2	105.00	C31A—C41A—H43A	109.00
C1A—C2A—H2A	120.00	H41A—C41A—H42A	109.00
C3A—C2A—H2A	119.00	H41A—C41A—H43A	109.00
C1B—C2B—H2B	119.00	H42A—C41A—H43A	109.00
C3B—C2B—H2B	119.00	C31B—C41B—H41B	109.00
C2A—C3A—H3A	120.00	C31B—C41B—H42B	109.00
C4A—C3A—H3A	120.00	C31B—C41B—H43B	110.00
C2B—C3B—H3B	120.00	H41B—C41B—H42B	109.00
C4B—C3B—H3B	120.00	H41B—C41B—H43B	110.00
C4A—C5A—H5A	120.00	H42B—C41B—H43B	109.00
			
C11A—O4A—C4A—C3A	−173.0 (2)	C6B—C1B—C2—C1	144.1 (2)
C11A—O4A—C4A—C5A	7.3 (3)	C6B—C1B—C2—C1A	−84.1 (2)
C4A—O4A—C11A—C21A	167.44 (19)	C2—C1B—C2B—C3B	−175.0 (2)
C11B—O4B—C4B—C3B	−170.0 (2)	C6B—C1B—C2B—C3B	1.9 (3)
C11B—O4B—C4B—C5B	10.1 (3)	C2—C1B—C6B—C5B	174.8 (2)
C4B—O4B—C11B—C21B	168.5 (2)	C2B—C1B—C6B—C5B	−2.5 (3)
Cl1—C1—C2—C1A	−179.73 (16)	C1A—C2A—C3A—C4A	0.0 (3)
Cl1—C1—C2—C1B	−47.6 (2)	C1B—C2B—C3B—C4B	0.9 (3)
Cl2—C1—C2—C1A	62.3 (2)	C2A—C3A—C4A—O4A	178.1 (2)
Cl2—C1—C2—C1B	−165.60 (16)	C2A—C3A—C4A—C5A	−2.2 (3)
Cl3—C1—C2—C1A	−57.1 (2)	C2B—C3B—C4B—O4B	176.8 (2)
Cl3—C1—C2—C1B	75.0 (2)	C2B—C3B—C4B—C5B	−3.3 (3)
C2A—C1A—C2—C1	89.6 (3)	O4A—C4A—C5A—C6A	−178.2 (2)
C2A—C1A—C2—C1B	−44.0 (3)	C3A—C4A—C5A—C6A	2.1 (3)
C6A—C1A—C2—C1	−91.4 (3)	O4B—C4B—C5B—C6B	−177.3 (2)
C6A—C1A—C2—C1B	135.1 (2)	C3B—C4B—C5B—C6B	2.8 (3)
C2—C1A—C2A—C3A	−178.6 (2)	C4A—C5A—C6A—C1A	0.4 (4)
C6A—C1A—C2A—C3A	2.4 (3)	C4B—C5B—C6B—C1B	0.1 (3)
C2—C1A—C6A—C5A	178.4 (2)	O4A—C11A—C21A—C31A	−62.3 (3)
C2A—C1A—C6A—C5A	−2.5 (4)	O4B—C11B—C21B—C31B	−63.9 (3)
C2B—C1B—C2—C1	−38.9 (3)	C11A—C21A—C31A—C41A	−177.8 (2)
C2B—C1B—C2—C1A	92.9 (3)	C11B—C21B—C31B—C41B	−171.1 (3)

### Hydrogen-bond geometry (Å, º)

**Table:**

*D*—H···*A*	*D*—H	H···*A*	*D*···*A*	*D*—H···*A*
C2*B*—H2*B*···Cl3	0.93	2.69	3.361 (2)	130
